# Hypoxic tumor-derived exosomal miR-4488 induces macrophage M2 polarization to promote liver metastasis of pancreatic neuroendocrine neoplasm through RTN3/FABP5 mediated fatty acid oxidation

**DOI:** 10.7150/ijbs.96831

**Published:** 2024-06-03

**Authors:** Feiyu Lu, Mujie Ye, Yikai Shen, Yanling Xu, Chunhua Hu, Jinhao Chen, Ping Yu, Bingyan Xue, Danyang Gu, Lin Xu, Lingyi Chen, Yi Ding, Jianan Bai, Ye Tian, Qiyun Tang

**Affiliations:** 1 Department of Geriatric Gastroenterology, Neuroendocrine Tumor Center, The First Affiliated Hospital of Nanjing Medical University, NO.300 Guangzhou Road, Nanjing 210029, Jiangsu Province, China.; 2 Department of General Surgery, The First Affiliated Hospital of Nanjing Medical University, NO.300 Guangzhou Road, Nanjing 210029, Jiangsu Province, China.

**Keywords:** pancreatic neuroendocrine neoplasms, M2-like polarization, hypoxia, exosomes, miR-4488, RTN3, FABP5, fatty acid oxidation, MMP2

## Abstract

Tumor-associated macrophages (TAMs) represent a predominant cellular component within the tumor microenvironment (TME) of pancreatic neuroendocrine neoplasms (pNENs). There is a growing body of evidence highlighting the critical role of exosomes in facilitating communication between tumor cells and TAMs, thereby contributing to the establishment of the premetastatic niche. Nonetheless, the specific mechanisms through which exosomes derived from tumor cells influence macrophage polarization under hypoxic conditions in pNENs, and the manner in which these interactions support cancer metastasis, remain largely unexplored. Recognizing the capacity of exosomes to transfer miRNAs that can modify cellular behaviors, our research identified a significant overexpression of miR-4488 in exosomes derived from hypoxic pNEN cells. Furthermore, we observed that macrophages that absorbed circulating exosomal miR-4488 underwent M2-like polarization. Our investigations revealed that miR-4488 promotes M2-like polarization by directly targeting and suppressing RTN3 in macrophages. This suppression of RTN3 enhances fatty acid oxidation and activates the PI3K/AKT/mTOR signaling pathway through the interaction and downregulation of FABP5. Additionally, M2 polarized macrophages contribute to the formation of the premetastatic niche and advance pNENs metastasis by releasing MMP2, thereby establishing a positive feedback loop involving miR-4488, RTN3, FABP5, and MMP2 in pNEN cells. Together, these findings shed light on the role of exosomal miRNAs from hypoxic pNEN cells in mediating interactions between pNEN cells and intrahepatic macrophages, suggesting that miR-4488 holds potential as a valuable biomarker and therapeutic target for pNENs.

## Introduction

pNENs represent a diverse and relatively rare category of tumors that originate from the endocrine islet cells of the pancreas, exhibiting neuroendocrine differentiation 1. As the second most prevalent form of pancreatic tumors, pNENs account for 3-5% of all pancreatic cancer cases. Data from the Surveillance, Epidemiology, and End Results (SEER) database indicate that the incidence of pNENs has experienced a fivefold increase in recent decades, outpacing the growth rate of other pancreatic cancer histologies 2. Despite a common misconception of pNENs being less aggressive, the clinical outlook for many patients is grim. More than 60% of pNEN patients are diagnosed with distant metastases, substantially affecting survival rates. The median overall survival for patients with localized pNENs is reported at 235 months, but this figure sharply declines to just 20 months for those with metastatic disease 3. Given that the mortality associated with pNENs primarily results from widespread metastasis, there is a pressing need to deepen our understanding of the molecular dynamics driving metastatic pNENs. Such insights are crucial for improving clinical outcomes through more informed therapeutic strategies and decision-making processes.

Hypoxia is a critical and complex component of the tumor microenvironment (TME), significantly contributing to tumor aggressiveness and the metastatic process 4. This condition arises from the imbalance between the high oxygen demand of rapidly proliferating cancer cells and the inadequate oxygen supply, which is often due to irregular tumor vascularization or the tumor's distance from supportive blood vessels 5. In response to hypoxic stress, cancer cells undergo extensive genetic reprogramming that fosters angiogenesis, epithelial-to-mesenchymal transition (EMT), and metastasis, enhancing cell survival and adaptability under low oxygen conditions 6. Recent research has underscored the significance of communication between tumor cells and their microenvironment in response to hypoxia, particularly through the secretion of exosomes by hypoxic tumor cells 7. Exosomes are small (30-150 nm) membrane-bound vesicles that carry diverse biomolecules, including various types of RNAs, DNAs, and proteins, with RNAs being identified as the primary bioactive components of these tumor-derived vesicles 8. These exosomes can transfer their contents to recipient cells, inducing phenotypic changes and facilitating intercellular communication 9. Evidence increasingly suggests that hypoxia stimulates the elevated production of exosomes, highlighting their role as crucial regulators within hypoxic tumors 10. By transferring genetic material to recipient cells, these exosomes can activate proliferative pathways, foster immune suppression, and establish premetastatic or metastatic niches 11. This understanding points to exosomes as not only key players in the adaptation and communication processes within the TME but also as potential targets for therapeutic intervention to disrupt the pathological interactions driving tumor progression and metastasis.

Macrophages play a pivotal role within TME, representing the most prevalent type of infiltrative immune-related stromal cells in and around tumors 12. These cells often adopt a protumoral M2-like phenotype, which is instrumental in fostering tumor initiation, progression, and metastasis 13. The functional reprogramming of macrophages into this phenotype is a multifaceted process that transcends the simple influence of IL-4, with the underlying mechanisms still largely unexplored. Recent studies have illuminated that macrophages undergo significant metabolic shifts—including alterations in lipid, glucose, and glutamine metabolism—and activate various signaling pathways to adapt to the unique conditions of their local TME 14. These adaptations endow them with immunosuppressive and anti-inflammatory capabilities, crucial for their role in tumor promotion. The interest in M2 macrophages has grown due to their capacity to release a plethora of cytokines such as TGF-β, TNF-α, IL-1β, IL-8, CCL2, CCL-5, ERO-1, MMP2, MMP3, MMP9, CSF-1, VEGF, and PLGF 15. These cytokines play significant roles in promoting metastasis by facilitating processes such as EMT and angiogenesis 16. This evidence underscores the critical function of M2 macrophages in tumor development and highlights the need for further research to fully understand their contributions to cancer progression and potential as therapeutic targets.

In our study, we discovered that exosomes derived from pNEN cells, under a hypoxic microenvironment, are enriched with miR-4488. These exosomes have the capability to polarize macrophages towards an M2-like phenotype by directly suppressing the expression of RTN3 in macrophages. Mechanistically, the downregulation of RTN3 in macrophages enhances fatty acid oxidation and activates the PI3K/AKT/ mTOR signaling pathway by interacting with and reducing the levels of FABP5. Interestingly, the M2-polarized macrophages contribute to the formation of a premetastatic niche and facilitate the metastasis of pNENs by secreting MMP2. This secretion triggers a miR-4488/RTN3/FABP5/MMP2 feedback loop in pNEN cells, highlighting a complex interplay between pNEN cells and the tumor microenvironment that promotes tumor progression and metastasis. This discovery not only elucidates a novel aspect of tumor-macrophage interaction but also opens new avenues for targeting the microenvironmental factors influencing cancer metastasis.

## Methods

### Cell culture and hypoxia treatment

Human pNEN cell line QGP-1 was obtained from the JCRB cell bank (JCRB0183) and grown in RPMI-1640 medium supplemented with 10% fetal bovine serum (FBS; Yeason, Shanghai, China) and 1% penicillin-streptomycin solution. Human pNEN cell line BON-1 was granted by Professor Xianjun Yu of Fudan University Shanghai Cancer Center. THP-1 cells were purchased from the Cell Bank of the Chinese Academy of Sciences and cultured in RPMI-1640 medium supplemented with 10% FBS. All cells were incubated in a humidified atmosphere of 5% CO2 and 95% air at 37°C. THP-1 cells were incubated with 100 ng/mL phorbol-12-myristate-13-acetate (PMA, Sigma, German) to induce differentiation into macrophages in vitro. For hypoxia treatment and exosome isolation, QGP-1 and BON-1 cells were cultured in a hypoxia incubator of 1% O_2_ with contained exosome-depleted FBS (SBI) medium.

### Exosome isolation and identification

QGP-1 and BON-1 cells were cultured in standard medium until reaching 80% confluency, at which point the medium was replaced with one containing 10% exosome-depleted FBS, under either normoxic or hypoxic (1% O2) conditions. After 48 hours, 15 mL of conditioned cell culture medium was harvested from each dish for exosome isolation. The isolation process involved sequential centrifugation of the conditioned medium: initially at 300×g for 10 minutes, then 2,000×g for 10 minutes, followed by 18,000×g for 30 minutes to eliminate cell fragments, debris, and larger extracellular vesicles. Subsequently, the supernatant was ultracentrifuged at 120,000×g for 70 minutes (Beckman Coulter). The resulting pellet was either resuspended in 50-100 μL PBS for immediate analysis or stored at -80°C for future use. The morphology and size of the isolated exosomes were examined using transmission electron microscopy (TEM, FEI Tecnai G2 12, USA). The ZetaView PMX 110 system (Particle Metrix, Germany) was employed to quantify the exosome concentration and total count.

### Western blotting

Total cellular protein was extracted using RIPA Lysis buffer (Beyotime, Shanghai, China), supplemented with a protease inhibitor (Roche, Mannheim, Germany). The protein samples underwent separation via 10% sodium dodecyl sulfate-polyacrylamide gel electrophoresis (SDS-PAGE) and were subsequently transferred to polyvinylidene fluoride (PVDF) membranes (Millipore, Billerica, USA). These membranes were then blocked using 5% bovine serum albumin (Sigma-Aldrich, St. Louis, USA), followed by overnight incubation at 4°C with primary antibodies (detailed in Supplementary Table S2). This was succeeded by a 1-hour room temperature incubation with horseradish peroxidase-conjugated secondary antibodies. Protein bands were visualized using Immobilon Western Chemiluminescent horseradish peroxidase Substrate (Millipore) and captured with the ChemiDoc XRS+ imaging system (Bio-Rad).

### Exosome labeling and tracking

Purified, isolated exosomes were collected and labeled according to the manufacturer's instructions, mixing them with Diluent C buffer and 4 μL PKH67 dye (Sigma, USA). The labeled exosomes were then resuspended in PBS and incubated with PMA-treated THP-1 cells at 37°C for 8 hours. Following incubation, the cells were stained with DAPI and analyzed using a laser scanning confocal microscope.

### Quantitative real-time PCR and RNA-seq

Total cellular RNA was isolated using TRIzol reagent (Life Technologies, USA), and exosomal RNA was extracted with a SeraMir Exosome RNA Extraction Kit (System Biosciences, USA). Subsequently, 1 µg of RNA was reverse-transcribed into complementary DNA (cDNA) utilizing the PrimeScript RT Reagent Kit (Yeason, China). Quantitative reverse transcription PCR (qRT-PCR) was conducted on an ABI PRISM 7900HT Sequence Detection System (Applied Biosystems, Waltham, MA, USA) employing ChamQ Universal SYBR qPCR Master Mix (Yeason, China). The expression levels of miR-4488 relative to U6 and gene expression relative to GAPDH were quantified using the comparative cycle threshold (2^-ΔΔCt^) method. Details regarding primers are provided in Table S1 of the supplementary material. For RNA sequencing, RNA samples were sequenced by Lianchuan Biotech (Hangzhou, China) and data were analyzed using OmicStudio tools available at https://www.omicstudio.cn/tool.

### Migration assay

1×10^5^ QGP-1 cells or 5×10^4^ BON-1 cells were suspended in 200 μL of serum-free medium and seeded in the upper chamber (diameter = 8 μm; Corning, USA). Simultaneously, THP-1 cells, either incubated with exosomes or transfected with plasmids, were added in 800 μL of medium containing 10% FBS to the lower chamber. After 24 hours of co-culture, the cells were stained with 0.1% crystal violet for 30 minutes. Non-migrating cells were then removed, and three random fields of view were selected for counting the migrated cells.

### Construction of stably transfected cell lines

miR-4488 inhibitors/mimics and negative controls, along with MMP2 siRNAs, were acquired from GenePharma Co. and transfected using Lipofectamine 3000 Transfection Reagent (Thermo Fisher Scientific, USA). Lentiviral vectors designed to express or suppress miR-4488 were produced by ViGene Biosciences Co. Additionally, Genomeditech was responsible for the construction of knockdown and overexpression plasmids for RTN3 and FABP5. Stable transfection was achieved following selection with puromycin, with confirmation by western blot. Detailed sequence information can be found in Table S3 of the supplemental material.

### Immunofluorescence (IF) staining

For IF staining on mouse liver tissue sections, the process began with deparaffinization and antigen retrieval using a retrieval solution (Beyotime Biotechnology, China) in a pressure cooker for 20 minutes. These steps were not required for IF staining on cryosections. Tissues were then blocked with 5% bovine serum albumin (BSA) in PBS containing 0.1% TWEEN 20 (PBS-T) for 30 minutes at room temperature. In the case of cultured cell IF staining, cells were fixed with 4% paraformaldehyde for 20 minutes, rinsed three times with PBS, and permeabilized with or without 0.1% Triton X-100 in PBS for 15 minutes. This was followed by a blocking step in 2% BSA in PBS-T for 30 minutes at room temperature. Next, samples were incubated with primary antibodies overnight at 4°C. Fluorophore-conjugated secondary antibodies were applied for 2 hours at room temperature. Filamentous actin was visualized using Phalloidin-488 (A12379, Invitrogen) or Phalloidin-555 (A34055, Invitrogen), and nuclei were counterstained with DAPI. Fluorescence images were acquired using a Stellaris STED laser scanning confocal microscopy system (LEICA, Germany). Details on the antibodies used are available in Supplementary Table S2.

### Nile red staining

For Nile red staining, 2×10^3^ exosome-incubated or plasmid-transfected PMA-treated THP-1 cells were seeded in glass bottom dishes (20mm, Cellvis, USA), After adherence, the cells were fixed with 4% paraformaldehyde for 20 min and then incubated with Nile Red working fluid (MCE, China) and Hoechst 33342 stain (Yeason, China) for 20 min at room temperature. Images were taken from random microscope fields.

### Flow cytometry

To identify CD206+ macrophages, THP-1 cells treated with PMA were initially harvested and fixed overnight in 1% paraformaldehyde (PFA) at 4°C. Subsequently, the cells were resuspended in flow cytometry buffer (1× PBS containing 1% FSA) and stained with anti-CD206 antibody (Invitrogen, USA) for 30 minutes at room temperature.

### Mass spectrometry

For mass spectrometry analysis, around 1×10^7^ THP-1 cells were lysed and incubated with either RTN3 antibody or normal IgG antibody for 4 hours at 4°C. Following this incubation, the samples were further incubated with 50μL of protein A/G agarose beads (Beyotime) at room temperature for 1 hour. Subsequently, the bead-probe-protein complexes were washed with wash buffer. The proteins bound to the beads were eluted by boiling in SDS buffer and then separated by SDS-PAGE. The protein bands specific to the RTN3 probe group, as opposed to those in the IgG group, were subjected to analysis using a Q Exactive mass spectrometer (Thermo Fisher Scientific, CA, USA).

### Co-immunoprecipitation

Equal quantities of THP-1 cells were treated with anti-RTN3, anti-FABP5, or anti-IgG antibodies, respectively. Following this, 50μL of protein A/G agarose beads (Beyotime) were introduced to each sample and incubated under rotation at room temperature for 1 hour. The bead-probe-protein complexes were subsequently washed with wash buffer and analyzed via western blotting.

### Haematoxylin and eosin (H&E) and immunohistochemical (IHC) staining

Mouse liver tissues were fixed in 4% paraformaldehyde, paraffin-embedded, and sectioned into 5 μm slices for H&E staining. For IHC staining, the liver tissues underwent fixation in 4% paraformaldehyde, dehydration through an ethanol series, and paraffin embedding. The tissues were then sectioned into 4 μm slices and treated with 3% H_2_O_2_ for 20 minutes to block endogenous peroxidase activity. After blocking with a suitable blocking solution for 30 minutes, the sections were incubated with the primary antibody overnight at 4°C. The following day, the sections were incubated with a secondary antibody for 1 hour at room temperature, followed by staining with diaminobenzidine (DAB) solution and hematoxylin for signal visualization. Imaging was performed using a Leica DM 2500 microscope (Germany).

### Animals

The animal experimental protocols for this study were thoroughly reviewed and received approval from the Animal Ethics Committee of Nanjing Medical University. All mice used in the study were sourced from the Laboratory Animal Centre of Nanjing Medical University and maintained in a specific pathogen-free environment. Four-week-old male athymic nude mice were randomly allocated into various groups, in alignment with the experimental design requirements. For the experiments involving exosome treatments, exosomes secreted by QGP-1 cells (subjected to different treatments), were resuspended in 200 μl of PBS and administered to the mice via tail vein injection. This procedure was repeated bi-daily over a span of two weeks. Following the conclusion of the exosome treatment period, a mixture of luciferase-labeled QGP-1 cells (1×10^6^ cells per mouse) and macrophages (5×10^5^ cells per mouse) was injected into the mice through the splenic vein. Three weeks subsequent to the spleen injection, the mice were subjected to in vivo imaging (Calliper Life Sciences, Hopkinton, MA, USA), after which they were euthanized to allow for the collection of their livers. The harvested livers were then processed for photography, H&E staining, and IF staining.

### Statistical analysis

All experiments were conducted with a minimum of three independent repetitions, and the results were expressed as the mean ± standard deviation (SD). Statistical analyses were performed using the paired two-tailed Student's t-test with GraphPad Software Inc. (La Jolla, CA, USA). A P value of less than 0.05 was deemed to indicate statistical significance.

## Results

### Hypoxia promotes exosomes secretion of pNEN cells to induce macrophages M2 polarization

**Figure 1:**
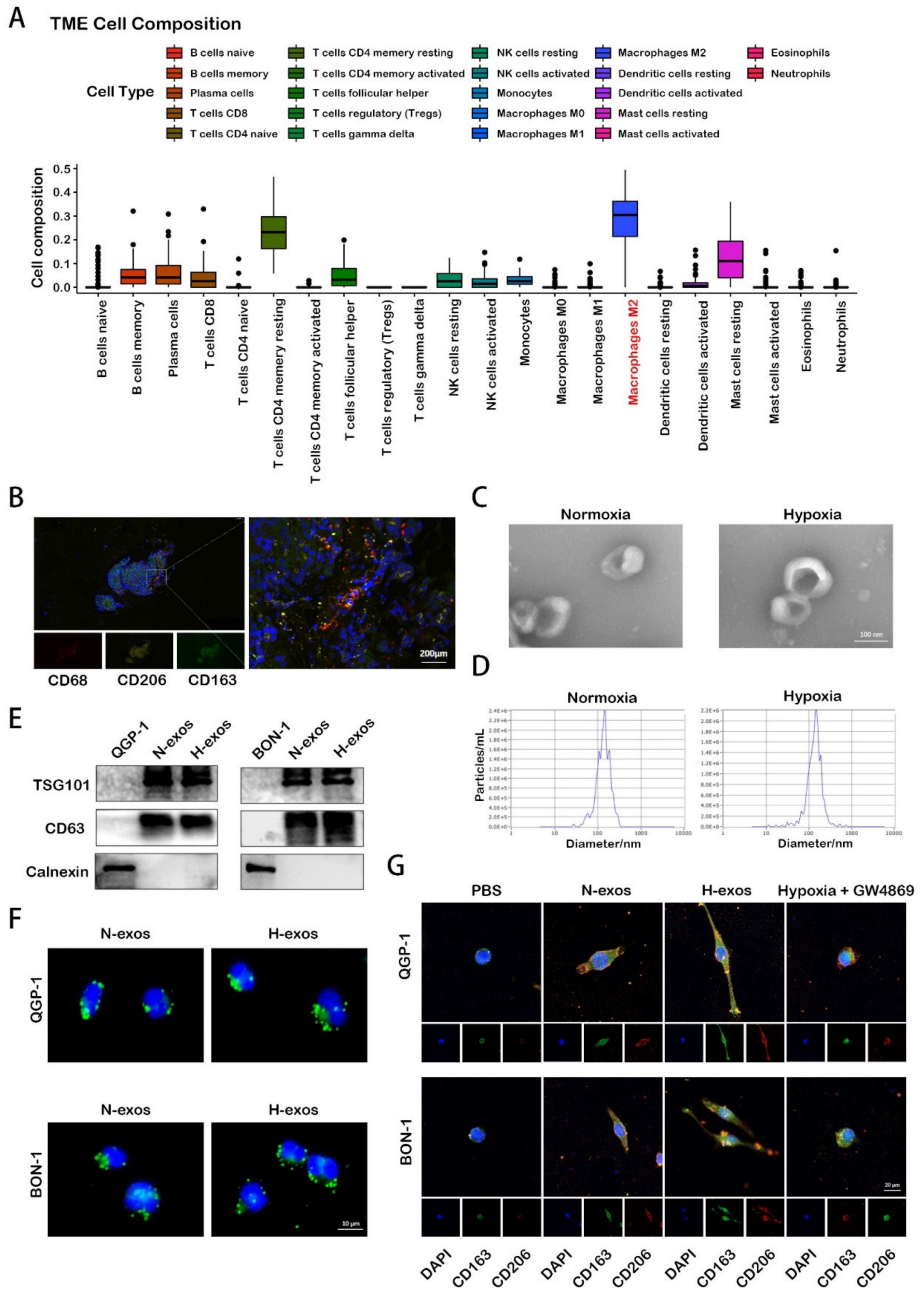
** Hypoxia promotes exosomes secretion of pNEN cells to induce macrophages M2 polarization.** A. The TME composition of pNENs were analyzed based on EGA database. B. IF image demonstrating the co-localization of CD68, CD206, CD163 in tissue of pNENs patient. C. Representative images of exosomes from normoxic and hypoxic QGP-1 cells at TEM. D. Purifed QGP-1-N-exos and QGP-1-H-exoswere analyzed by NanoSight. E. Western blot analysis of exosome markers. F. Representative IF image showing internalization of PKH67-labeled exosomes (green) by PMA-treated THP-1 cells. G. IF image demonstrating the expression of CD206 and CD163 in macrophages treated with PBS, N-exos, H-exos or exosomes from pNEN cells treated with GW4869 under hypoxia.

Macrophages play a crucial role in tumor initiation, progression, and metastasis. Our analysis, leveraging data from the European Genome-phenome Archive (EGA), reveals that M2 polarized macrophages are the predominant infiltrative immune-related stromal cells in pNENs (Fig. 1A). Immunofluorescence analysis of human pNEN tissues highlighted extensive co-localization of the markers CD68, CD206, and CD163 (Fig. 1B), underscoring their significance in the TME. Given the complex role of hypoxia in the TME— notably, its promotion of cell-cell communication through enhanced exosome secretion, the adequacy of hypoxia treatment was initially verified by assessing the expression of hypoxia-inducible factor-1α (HIF-1α) using Western blot analysis (Fig. S1A). Subsequently, exosomes were isolated from the conditioned media after 48 hours. These exosomes were quantified using electron microscopy and nanoparticle tracking analysis (Fig. 1C, 1D). Western blot analysis further confirmed the presence of exosomal proteins TSG101 and CD63 in these isolates (Fig. 1E). Subsequent experiments assessed the impact of hypoxic exosomes on macrophage polarization. When co-cultured with PMA-treated macrophages, tumor-derived hypoxic exosomes, marked with PKH67 fluorescent dye, were internalized by the macrophages over time (Fig. 1F). Immunofluorescence analysis further revealed that exposure to hypoxic exosomes significantly increased the expression of CD206 and CD163 in macrophages compared to those treated with normoxic exosomes or PBS. This effect was diminished by the inhibition of exosome secretion with GW4869 (Fig. 1G). These findings indicate that hypoxia-induced exosome secretion from pNEN cells notably promotes the activation of macrophages towards an M2 phenotype, emphasizing the intricate interplay between hypoxia and immune modulation within the TME.

### M2 macrophages induced by hypoxic exosomes promote the metastasis of pNEN cells

**Figure 2:**
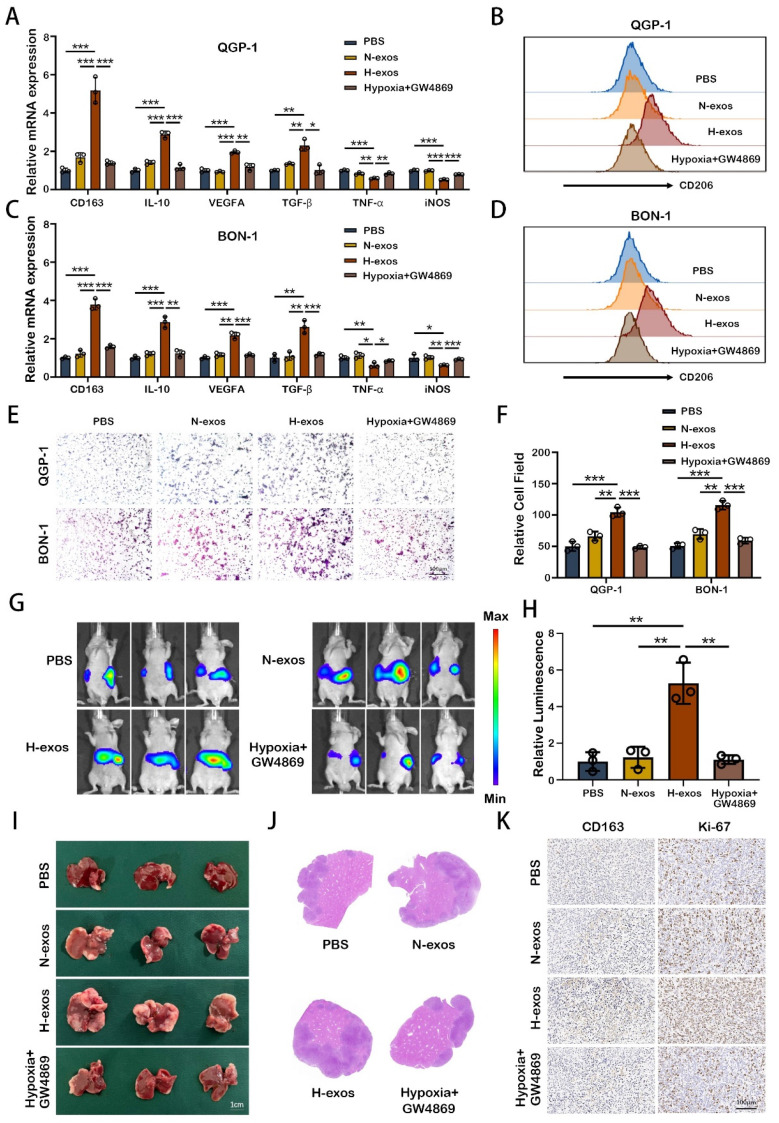
** M2 macrophages induced by hypoxic exosomes promote the metastasis of pNEN cells.** A. The expression of typical M2 markers (CD163, IL10, VEGFA and TGF-β) and M1 markers (TNF-α and iNOS) in PMA-treated THP-1 cells incubated with PBS, QGP-1-N-exos, QGP-1-H-exos or exosomes from QGP-1 cells treated with GW4869 under hypoxia were examined by qRT-PCR. B. The effect of QGP-1 cell-derived exosomes on the expression of CD206 (M2 marker) was detected by flow cytometry. C. The expression of typical M2 markers and M1 markers in PMA-treated THP-1 cells incubated with PBS, BON-1-N-exos, BON-1-H-exos or exosomes from BON-1 cells treated with GW4869 under hypoxia were examined by qRT-PCR. D. The effect of BON-1 cell-derived exosomes on the expression of CD206 was detected by flow cytometry. E, F. Transwell assays evaluated the migration ability of pNEN cells cocultured with macrophages treated with PBS, N-exos, H-exos or exosomes from pNEN cells treated with GW4869 under hypoxia. G, H. Representative in vivo imaging system results of mice injected with macrophages and luciferase-labeled QGP-1 cells into the spleen after treated with PBS, QGP-1-N-exos, QGP-1-H-exos or exosomes from QGP-1 cells treated with GW4869 under hypoxia, respectively. Results of quantified values of bioluminescence imaging signals are expressed as mean±standard deviation. I, J. Effect of QGP-1-derived exosomes on liver metastasis in mice. The representative photographs of liver metastasis and H&E staining were shown. K. Representative pictures of IHC staining of CD163 and Ki-67 are shown. *P<0.05; **P<0.01; ***P<0.001.

To substantiate the influence of exosomes from hypoxic pNEN cells on macrophage polarization, we employed qRT-PCR. The results demonstrated that exosomes from hypoxic QGP-1 cells (QGP-1-H-exo) markedly upregulated the expression of M2 macrophage markers (CD163, IL-10, VEGFA, and TGF-β) and concurrently downregulated M1 macrophage markers (iNOS and TNF-α) in PMA-treated THP-1 cells, compared to treatments with PBS, normoxic exosomes (QGP-1-N-exo), or exosomes from QGP-1 cells treated with GW4869 (an exosomes inhibitor) under hypoxic conditions (Fig. 2A). Flow cytometry analysis further confirmed the significant enhancement of CD206 expression by QGP-1-H-exo in PMA-treated THP-1 cells (Fig. 2B). Similarly, exosomes from hypoxic BON-1 cells (BON-1-H-exo) significantly increased the expression of the same M2 markers and decreased the expression of M1 markers, compared to PBS, BON-1-N-exo, or exosomes from BON-1 cells treated with GW4869 under hypoxic, with corresponding enhancement in CD206 expression as evidenced by flow cytometry (Fig. 2C, 2D). Migration assays revealed that pNEN cells co-cultured with macrophages pre-treated with hypoxic exosomes exhibited a significant increase in migration ability compared to other groups (Fig. 2E, 2F), suggesting the role of M2 macrophages in promoting pNEN cell migration. To explore the potential for liver metastasis, the most common metastasis site for pNENs, an in vivo liver metastasis model was established.

Mice were intravenously injected with PBS, QGP-1-N-exo, QGP-1-H-exo, or exosomes from cells treated with GW4869 under hypoxia, followed by spleen injection of luciferase-labeled QGP-1 cells mixed with THP-1 cells. After three weeks, the strongest fluorescence signal was observed in mice treated with QGP-1-H-exo, indicating a significant increase in liver fluorescence intensity compared to other groups (Fig. 2G, 2H). Subsequent H&E staining of liver sections revealed a significant increase in the size and number of liver metastases in the QGP-1-H-exo group (Fig. 2I, 2J). Immunohistochemical staining showed elevated expression of CD163 and Ki-67 in mice treated with QGP-1-H-exo (Fig. 2K), underscoring the contribution of hypoxic pNEN cell-derived exosomes to M2 macrophage polarization and the establishment of an immunosuppressive tumor microenvironment (TME). This fosters the malignant progression of pNENs, highlighting the critical role of exosome-mediated intercellular communication in cancer metastasis.

### Hypoxic pNEN cells derived miR-4488 promotes M2 macrophage polarization in vitro and in vivo

**Figure 3:**
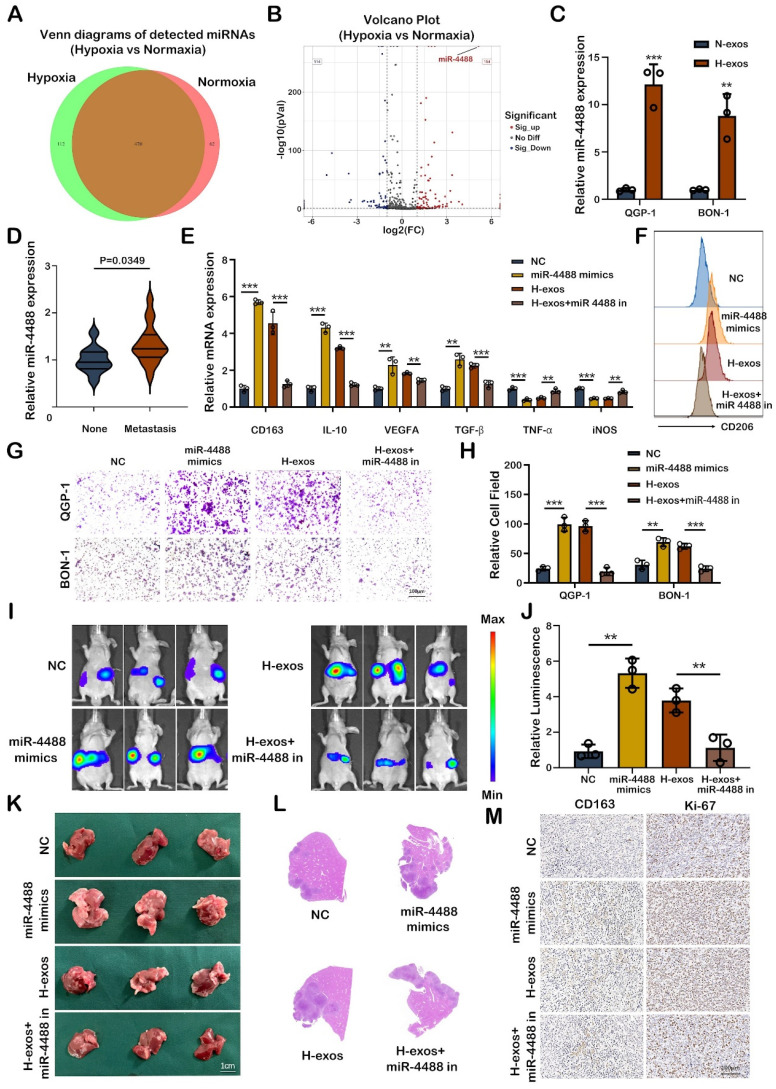
** Hypoxic pNEN cells derived miR-4488 promotes M2 macrophage polarization in vitro and in vivo.** A, B. Venn diagram and volcano plot showing the differential miRNAs expressed between QGP-1-N-exos and QGP-1-H-exos. C. The expression of miR-4488 expression in exosomes derived by normoxic or hypoxic pNEN cells were detected by qRT-PCR. D. The miR-4488 expression level in the serum of pNEN patients with metastasis compared to those without. E. The expression of typical M2 markers and M1 markers in PMA-treated THP-1 cells treated with mimics NC, miR-4488 mimics, H-exos or both H-exos and miR-4488 inhibitor were measured by qRT-PCR. F. The effect of miR-4488 on the expression of CD206 was detected by flow cytometry. G, H. Transwell assays evaluated the migration ability of pNEN cells cocultured with macrophages treated with mimics NC, miR-4488 mimics, H-exos or both H-exos and miR-4488 inhibitor. I, J. Representative in vivo imaging system results of mice injected with macrophages and luciferase-labeled QGP-1 cells into the spleen after treated with mimics NC, miR-4488 mimics, H-exos or both H-exos and miR-4488 inhibitor, respectively. Results of quantified values of bioluminescence imaging signals are expressed as mean±standard deviation. K, L. Effect of miR-4488 on liver metastasis in mice. The representative photographs of liver metastasis and H&E staining were shown. M. Representative pictures of IHC staining of CD163 and Ki-67 are shown. **P<0.01; ***P<0.001.

The miRNA content of exosomes plays a crucial role in intercellular communication, particularly in the context of cancer metastasis. In our study, exosomes derived from the supernatant of both normoxic and hypoxic QGP-1 cells were subjected to miRNA sequencing to identify miRNAs implicated in tumor metastasis. Venn diagram analysis identified 112 unique miRNAs differentially expressed between the exosomes from normoxic and hypoxic QGP-1 cells, with miR-4488 being significantly upregulated in the exosomes from hypoxic QGP-1 cells (Fig. 3A, 3B). Although miRNAs have been widely studied for their roles in various diseases, the specific regulatory function of miR-4488 in tumor progression remains underexplored. Further validation confirmed that miR-4488 was significantly overexpressed in the exosomes from hypoxic QGP-1 and BON-1 cells compared to their normoxic counterparts (Fig. 3C). Additionally, miR-4488 levels were found to be significantly elevated in the serum of pNEN patients with metastasis compared to those without (Fig. 3D). Given the established role of hypoxic pNEN cell-derived exosomes in promoting M2-like macrophage polarization and thereby facilitating metastasis, our investigation focused on whether miR-4488 mediates this process. pNEN cells transfected with miR-4488 mimics, and subsequently isolated exosomes (miR-4488-exos), were used to treat macrophages. The treatment with miR-4488-exos resulted in a significant upregulation of M2 macrophage markers and downregulation of M1 markers in PMA-treated THP-1 cells, as evidenced by qRT-PCR. This effect was in contrast to treatments with a non-coding (NC) vector, and the M2 polarization induced by QGP-1-H-exos was reversed upon co-transfection with a miR-4488 inhibitor (Fig. 3E). Flow cytometry analysis further confirmed the upregulation of CD206 expression in macrophages treated with miR-4488-exos compared to those treated with the NC vector, and this upregulation was also reversed by the miR-4488 inhibitor (Fig. 3F). Migration assays demonstrated a significant increase in the migration of pNEN cells co-cultured with macrophages pre-treated with miR-4488 mimics compared to controls. This carcinogenic effect was mitigated upon co-transfection with the miR-4488 inhibitor (Fig. 3G, 3H). In vivo liver metastasis models further showed that mice treated with miR-4488-exos or QGP-1-H-exos exhibited more extensive and larger liver metastases, as well as stronger fluorescence signals, compared to those treated with the NC vector or a combination of QGP-1-H-exos and the miR-4488 inhibitor (Fig. 3I-L). Immunohistochemical staining of liver sections from these mice revealed higher expression levels of CD163 and Ki-67, markers associated with macrophage activation and proliferation, respectively (Fig. 3M). Collectively, these findings illuminate the pivotal role of exo-miR-4488 in promoting liver metastasis of pNENs through the induction of M2 macrophage polarization, highlighting the significance of exosomal miRNAs in the modulation of the tumor microenvironment and metastatic progression.

### Exo-miR-4488 induces M2 macrophage polarization by targeting RTN3

**Figure 4:**
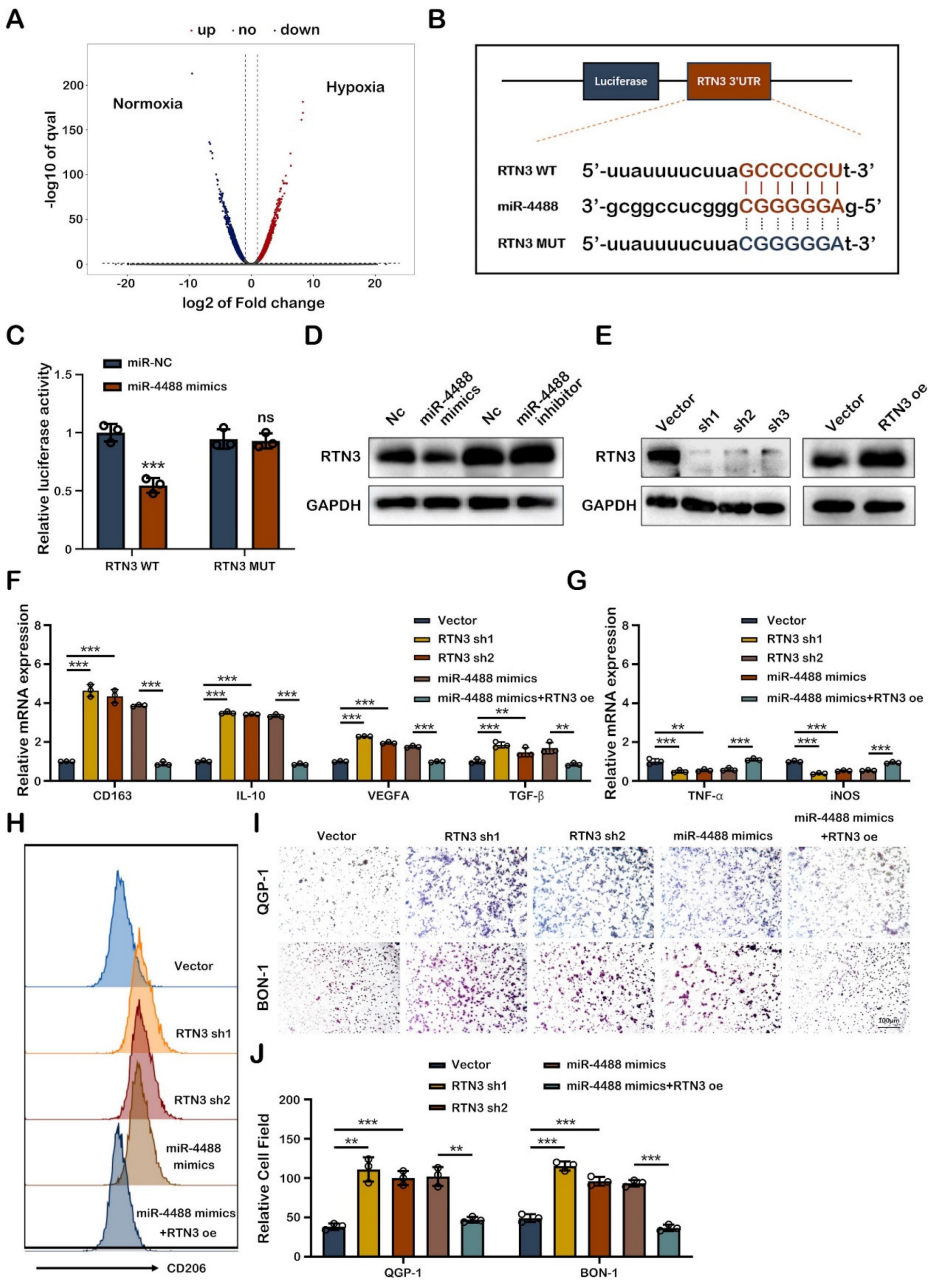
** Exo-miR-4488 induces M2 macrophage polarization by targeting RTN3.** A. volcano plot showing the differential mRNAs expressed between macrophages treated with QGP-1-N-exos and QGP-1-H-exos. B. Schematic representation of the wild-type and mutant-type binding site between the 3'UTR of RTN3 and miR-4488. C. Relative luciferase activity of 3'UTR-RTN3-luc constructs after transfection of miR-4488 mimics/NC. D. Expression of RTN3 in PMA-treated THP-1 cells in which miR-4488 was overexpressed or knocked down was detected by Western blot. E. Stable RTN3 knockdown or overexpression THP-1 cells were constructed. F, G. The expression of typical M2 markers and M1 markers in PMA-treated THP-1 cells transfected with NC Vector, RTN3 knockdown plasmid, miR-4488 mimics or both miR-4488 mimics and RTN3 overexpression plasmid were measured by qRT-PCR. H. The effect of RTN3 on the expression of CD206 was detected by flow cytometry. I, J. Transwell assays evaluated the migration ability of pNEN cells cocultured with macrophages transfected with NC Vector, RTN3 knockdown plasmid, miR-4488 mimics or both miR-4488 mimics and RTN3 overexpression plasmid. **P<0.01; ***P<0.001.

To elucidate how tumor-derived exosomal miR-4488 fosters M2 macrophage polarization, we focused on identifying the specific target of miR-4488 within macrophages. Differential mRNA expression in PMA-treated THP-1 cells co-cultured with QGP-1-N-exos and QGP-1-H-exos exosomes was examined through RNA sequencing, with the findings illustrated in a volcano plot (Fig. 4A). RTN3 emerged as a potential target of miR-4488, as predicted by TargetScan and miRDB, based on its 3' untranslated region (3'UTR) containing a putative binding site for miR-4488, as indicated by bioinformatic analysis (Fig. 4B). Luciferase reporter assays confirmed the direct interaction between miR-4488 and RTN3. Specifically, luciferase activity significantly decreased in cells co-transfected with miR-4488 mimics and a wild-type RTN3 construct (RTN3-WT), whereas no significant change was observed in cells transfected with a mutated RTN3 construct (RTN3-MUT), underscoring the specificity of miR-4488 targeting RTN3 (Fig. 4C). Western blot analysis revealed that transfection of miR-4488 mimics into PMA-treated THP-1 cells suppressed RTN3 expression.

Conversely, the effect of miR-4488 inhibitors on increasing RTN3 expression was minimal, possibly due to the low baseline expression of miR-4488 (Fig. 4D). Further experiments investigated RTN3's role in macrophage phenotype conversion. Western blotting confirmed the efficiency of RTN3 knockdown and overexpression (Fig. 4E). qRT-PCR showed that RTN3 knockdown significantly elevated M2 marker expression while reducing M1 marker expression. Moreover, the M2 polarization effect induced by miR-4488 mimics was reversed upon co-transfection with an RTN3 overexpression plasmid (Fig.4F, 4G). Flow cytometry demonstrated an increase in CD206 expression in PMA-treated THP-1 cells following RTN3 knockdown, an effect reversed by RTN3 overexpression, indicating the regulatory role of RTN3 in M2 polarization (Fig. 4H). Migration assays utilizing a co-culture system further assessed RTN3's impact on pNEN cell metastatic capability. The assays indicated a significant increase in the migration of pNEN cells co-cultured with macrophages post-RTN3 knockdown, an effect mitigated by co-transfection with an RTN3 overexpression plasmid (Fig. 4I, 4J). Collectively, these findings highlight the critical role of miR-4488 in targeting RTN3 to induce M2 macrophage polarization and subsequently promote the progression of pNENs.

### RTN3 interacted with FABP5 to increase fatty acid oxidation in TAMs

**Figure 5:**
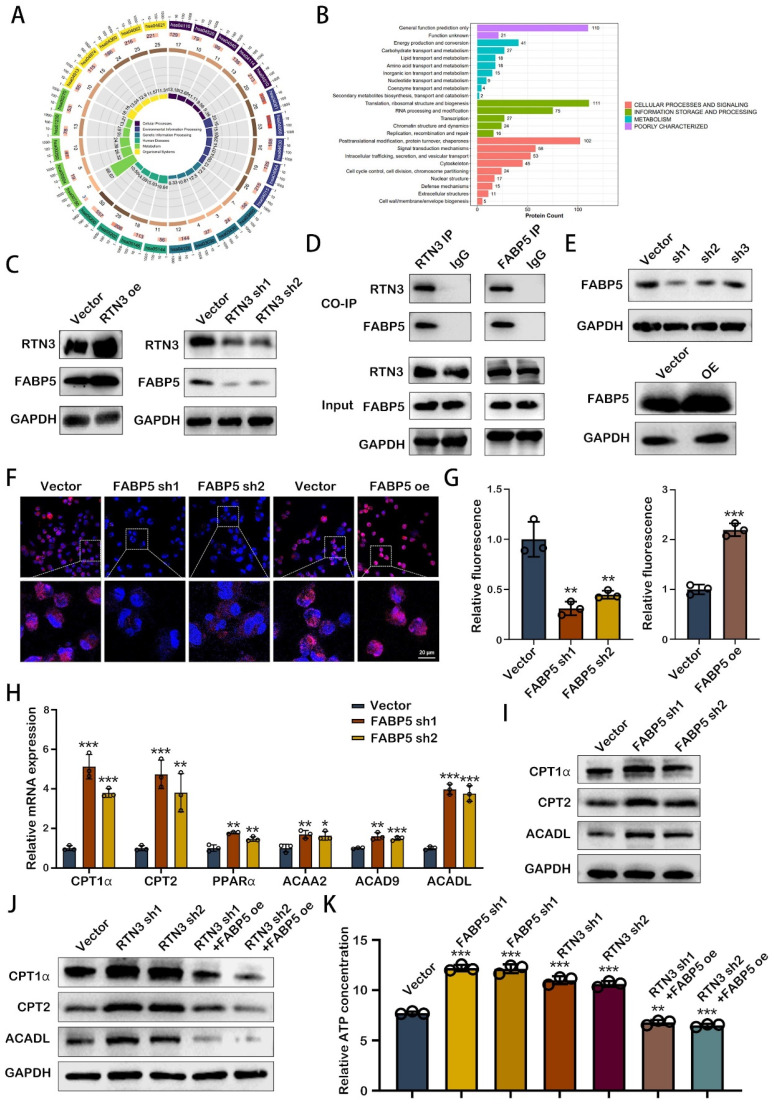
** RTN3 interacted with FABP5 to increase fatty acid oxidation in TAMs.** A. KEGG enrichment analysis of the differential genes induced by RTN3 knockdown. B. Enrichment analysis based on the proteins binding to RTN3. C. The effects of RTN3 overexpression and knockdown on FABP5 expression was detected by western blot. D. The interaction between RTN3 and FABP5 was demonstrated by co-immunoprecipitation. E. Stable FABP5 knockdown or overexpression THP-1 cells were constructed. F, G. Fluorescence images showing Nile red-stained oil droplets in PMA-treated THP-1 cells with RTN3 knockdown and overexpression. H. Expression of key markers of FAO were detected by qRT-PCR. I, J. The expression of CPT1α, CPT2 and ACADL were measured by western blot. K. Relative ATP concentration was measured in macrophages transfected with FABP5 knockdown, RTN3 knockdown or both FABP5 knockdown and RTN3 overexpression plasmids. **P<0.01; ***P<0.001.

Exploring the mechanism through which RTN3 modulates the phenotype of TAMs unveils a critical area of research, particularly focusing on energy metabolism's role in M2 macrophage polarization. Kyoto Encyclopedia of Genes and Genomes (KEGG) pathway analysis, upon RTN3 knockdown, highlighted the involvement of several genes in cellular metabolism pathways (Fig. 5A). Additionally, mass spectrometry analysis identified a subset of genes associated with RTN3 that play roles in lipid transport and metabolism (Fig. 5B). Among these, FABP5, a member of the fatty acid-binding proteins family, was found to interact with RTN3. Western blot analysis demonstrated that FABP5 expression increased with RTN3 overexpression and decreased with RTN3 knockdown, suggesting a regulatory relationship between RTN3 and FABP5 (Fig. 5C). This interaction was further validated through co-immunoprecipitation in THP-1 cells (Figure 5D), establishing a direct link between RTN3 and FABP5. To further determine the role of H-exos via the RTN3/FABP5 axis, we performed western blot analysis and found that H-exos could simultaneously inhibit the expression of FABP5 and RTN3 (Fig. S2A). To investigate the role of RTN3 in lipid metabolism, THP-1 cells with stable knockdown and overexpression of FABP5 were created, and their efficiency was confirmed via western blotting (Fig. 5E). Nile red staining indicated that intracellular lipid droplet quantity and intensity decreased with FABP5 knockdown, while FABP5 overexpression led to increased lipid droplet deposition (Fig. 5F, 5G), suggesting FABP5's involvement in lipid droplet accumulation. Given the connection between lipid droplet degradation and fatty acid oxidation (FAO), we examined FAO markers in FABP5-knockdown THP-1 cells using qRT-PCR. The results revealed significant upregulation of FAO markers, including CPT1α, CPT2, PPARα, ACAA2, ACAD9, and ACADL, particularly CPT1α, CPT2, and ACADL (Fig. 5H), which was corroborated by western blotting (Figure 5I). Further analysis showed that RTN3 could enhance the expression of these FAO markers, an effect that was reversed by FABP5 overexpression (Fig. 5J). This suggests a regulatory axis where RTN3 influences FAO through FABP5. ATP assays conducted to assess the functional consequences of these molecular interactions showed that knockdown of FABP5 or RTN3 increased ATP levels in THP-1 cells, indicating enhanced FAO. Conversely, FABP5 overexpression could negate the ATP level increase induced by RTN3 knockdown (Fig. 5K). These findings suggest that RTN3, by interacting with FABP5, promotes fatty acid oxidation in TAMs, highlighting a novel pathway through which RTN3 influences TAM metabolism and, consequently, tumor progression.

### RTN3 suppression promotes M2 macrophage polarization in a manner dependent on the PI3K/AKT/mTOR pathway

**Figure 6:**
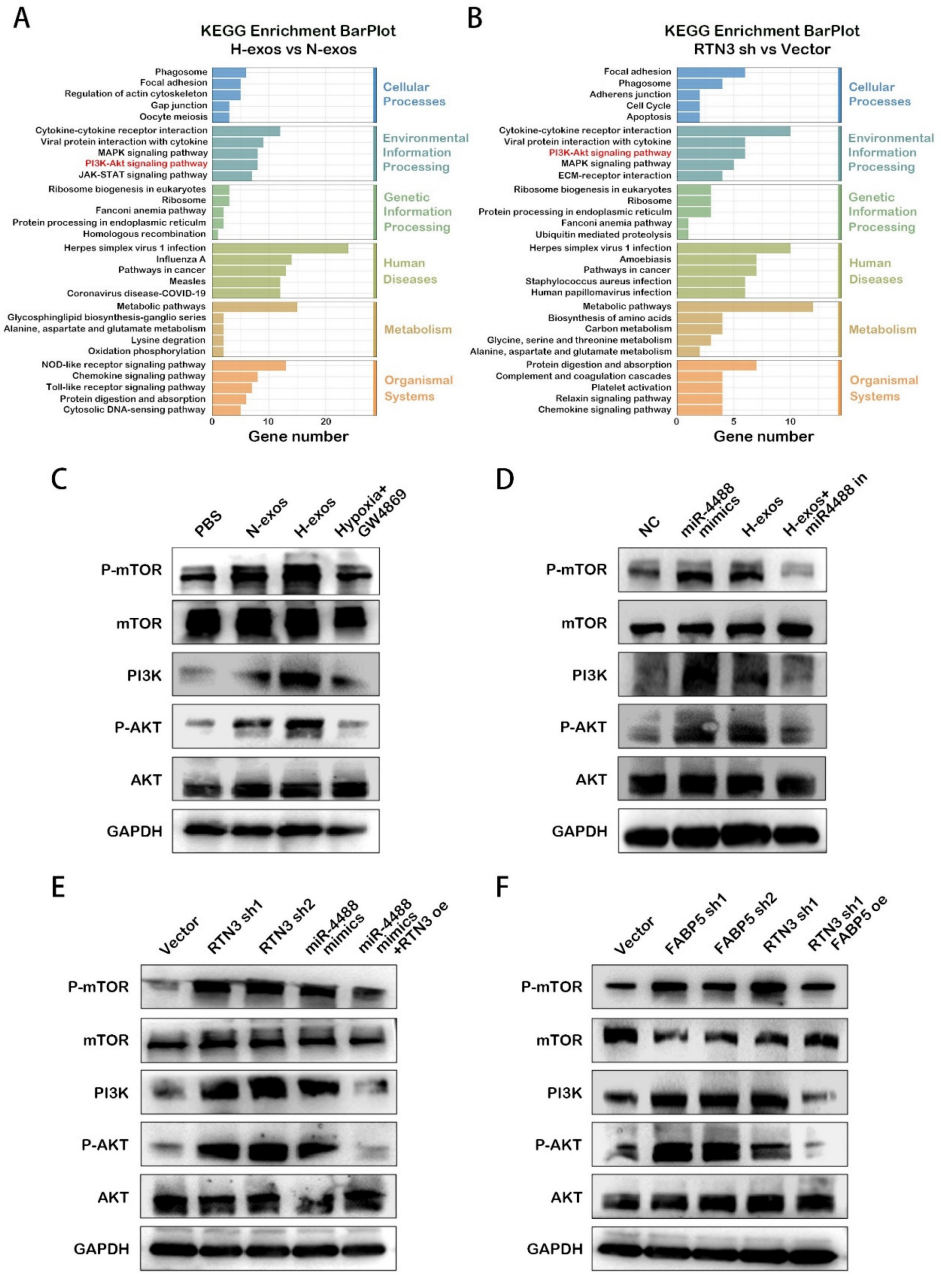
** RTN3 suppression promotes M2 macrophage polarization in a manner dependent on the PI3K/AKT/mTOR pathway.** A, B. KEGG enrichment analysis of the differential genes induced by RTN3 knockdown contrast to Vector NC or QGP-1-H-exos contrast to QGP-1-N-exos. C. Western blot results for the expression of proteins in the PI3K/AKT/mTOR signaling pathway in macrophages treated with PBS, QGP-1-N-exos, QGP-1-H-exos or exosomes from QGP-1 cells treated with GW4869 under hypoxia. D. Expression of proteins in the PI3K/AKT/mTOR signaling pathway in macrophages transfected with mimics NC, miR-4488 mimics, H-exos or both H-exos and miR-4488 inhibitor were measured by western blot. E. PI3K/AKT/mTOR signaling pathway related genes expression in macrophages transfected with NC Vector, RTN3 knockdown plasmid, miR-4488 mimics or both miR-4488 mimics and RTN3 overexpression plasmid were measured by western blot. F. Western blot was performed to detect the PI3K/AKT/mTOR signaling pathway related genes expression in macrophages transfected with NC Vector, FABP5 knockdown, RTN3 knockdown, or both FABP5 knockdown and RTN3 overexpression plasmids.

The PI3K/AKT/mTOR signaling pathway is well-established as a critical regulator of macrophage phenotypic conversion, with its activation influencing the metabolic pattern of these cells. KEGG pathway analysis of differentially expressed genes in macrophages treated with QGP-1-N-exos versus QGP-1-H-exos, as well as RTN3 knockdown macrophages compared to controls, showed enrichment in the activation of the PI3K/AKT/mTOR pathway (Fig. 6A, 6B). This suggests a potential mechanism by which suppression of RTN3 may facilitate M2 macrophage polarization through this pathway.

To explore this hypothesis, we conducted western blot analyses to examine the activation state of the PI3K/AKT/mTOR pathway. The results confirmed that QGP-1-H-exos treated macrophages exhibited activation of the PI3K/AKT/mTOR pathway (Fig. 6C). Similar activation was observed in PMA-treated THP-1 cells overexpressing miR-4488, with the effect being reversible by a miR-4488 inhibitor, implicating miR-4488 in the activation process induced by QGP-1-H-exos (Fig. 6D). Further analysis revealed that knockdown of RTN3 and FABP5 also resulted in activation of the PI3K/AKT/mTOR pathway. Rescue experiments demonstrated that overexpression of RTN3 could partially attenuate the effect of miR-4488 overexpression (Fig. 6E), and overexpression of FABP5 was capable of reversing the activation effect induced by RTN3 knockdown (Fig. 6F). These findings collectively underscore the critical role of RTN3 in the regulation of M2 macrophage polarization by hypoxic pNEN cell-derived exosomes. The process appears to be mediated via the activation of the PI3K/AKT/mTOR pathway, establishing a novel link between RTN3 suppression, miR-4488 expression, and macrophage phenotypic conversion. This mechanism highlights the complex interplay between genetic regulation, metabolic reprogramming, and signaling pathway activation in the context of tumor microenvironment modulation and provides valuable insights into potential therapeutic targets for influencing tumor-associated macrophage behavior and, consequently, tumor progression.

### M2 macrophage induced by RTN3 suppression facilitates the liver metastasis of pNEN cells via secreting MMP2

**Figure 7:**
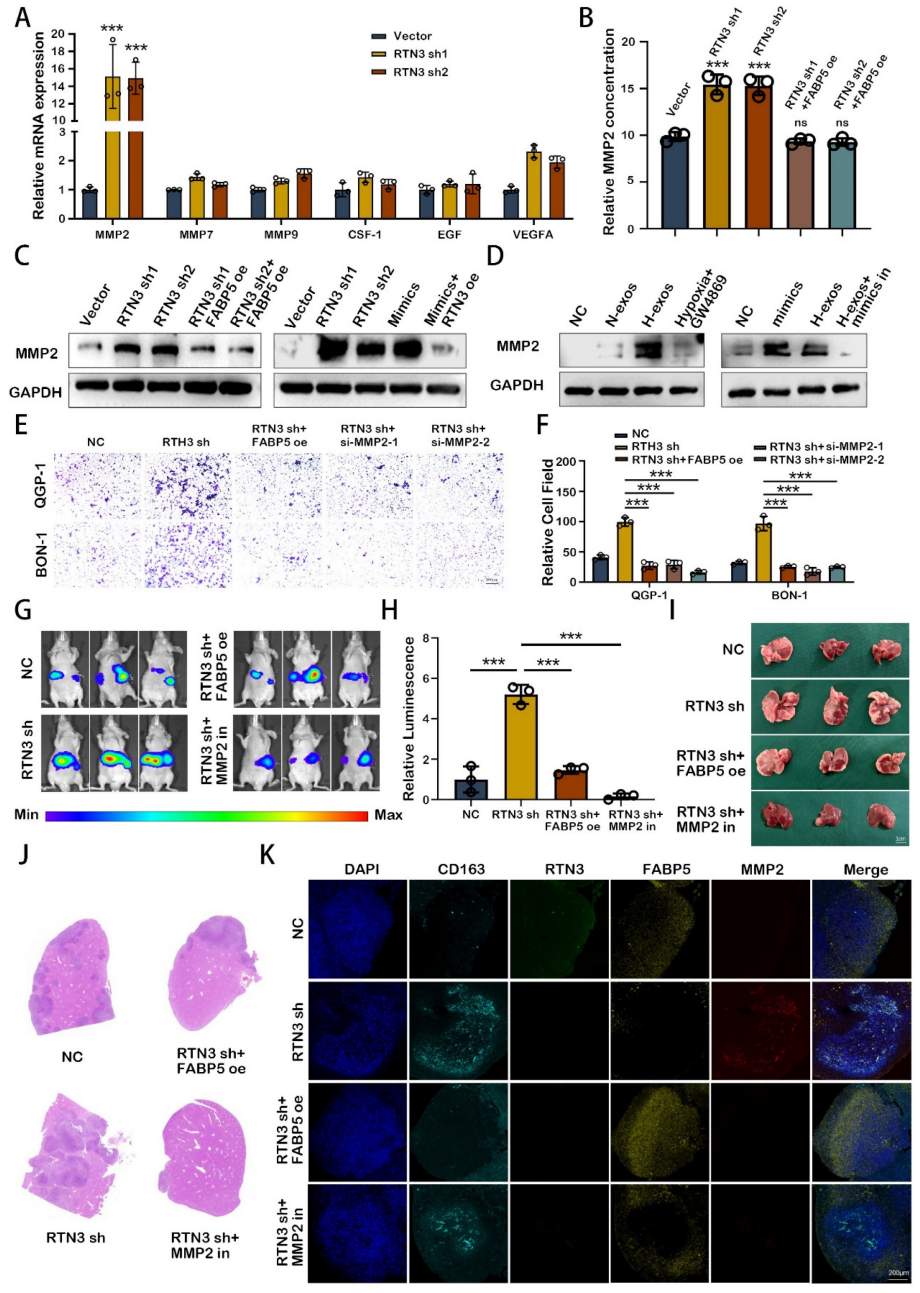
** M2 macrophage induced by RTN3 suppression facilitates the liver metastasis of pNEN cells via secreting MMP2.** A. The expression of key cytokines secreted by M2 macrophages induced by RTN3 knockdown was measured by qRT-PCR. B. The relative MMP2 concentration on supernatants from PMA-treated THP-1 cells transfected with RTN3 knockdown or both RTN3 knockdown and FABP5 overexpression plasmids was detected by ELISA assays. C, D. Western blot showed the effects of RTN3, FABP5, miR-4488 and QGP-1-H-exos on the expression of MMP2 in macrophages. E, F. Transwell assays evaluated the migration ability of pNEN cells cocultured with macrophages transfected with RTN3 knockdown, co-transfection with RTN3 knockdown and FABP5 overexpression and co-transfection with RTN3 knockdown and si-MMP2. G, H. Representative in vivo imaging system results of mice injected with RTN3 knockdown macrophages and luciferase-labeled QGP-1 cells into the spleen with or without treatment of MMP2 inhibitor. I, J. The representative photographs of liver metastasis and H&E staining were shown. K. IF image demonstrating the expression of CD163, RTN3, FABP5 and MMP2 in hepatic metastatic tissues of mice. *P<0.05; **P<0.01; ***P<0.001.

To elucidate the role of M2 polarized macrophages in the metastasis of pNENs, we assessed the expression of key cytokines via qRT-PCR. Our findings revealed a marked upregulation of matrix metalloproteinase-2 (MMP2) in macrophages with reduced RTN3 expression (Fig. 7A). Further analysis using ELISA on supernatants from PMA-treated THP-1 cells, subjected to conditional transfection, demonstrated that RTN3 suppression elevates MMP2 secretion, an effect mitigated by co-transfecting with FABP5 overexpression plasmid (Fig. 7B). Western blot analysis confirmed the increased MMP2 expression in RTN3-suppressed, PMA-treated THP-1 cells. Rescue experiments indicated that co-transfection with an RTN3 overexpression plasmid could counteract the effects of miR-4488 mimics, while FABP5 overexpression plasmid co-transfection could diminish the impact of RTN3 suppression (Fig. 7C). Additionally, treatment with QGP-1-H-exos augmented MMP2 expression, which was partially reduced by co-transfection with a miR-4488 inhibitor (Fig. 7D). To ensure that miR-4488, RTN3, and FABP5 do not independently affect the expression of MMP2 in tumor cells, we conducted Western blot experiments. The results showed that transfection with miR-4488 mimics, as well as knockdown plasmids for RTN3 and FABP5, did not alter MMP2 expression in pNEN cells (Fig. S3A). Transwell migration assays illustrated that the migratory capability of pNENs cells, enhanced by co-culture with RTN3-suppressed macrophages, was diminished by either co-transfection with an si-MMP2 or FABP5 overexpression plasmid, or by the application of an MMP2 inhibitor (Fig. 7E, 7F). In vivo, liver metastasis models demonstrated increased fluorescence signaling and a higher incidence and size of liver metastases in mice injected via the splenic vein with QGP-1 cells and RTN3-suppressed THP-1 cells. These effects were mitigated when macrophages were co-transfected with RTN3 and FABP5 overexpression plasmids or when mice were treated with MMP2-IN-1 (MedChem Express, China) at a dose of 5mg/kg/day (Fig. 7G-J). Immunofluorescence assays showed that FABP5 overexpression could effectively counteract the RTN3 suppression-induced M2 polarization, thereby inhibiting the metastasis of QGP-1 cells. Moreover, MMP2 inhibition through MMP2-IN-1 treatment also prevented metastasis facilitated by RTN3-suppressed macrophages (Fig. 7K). These findings suggest MMP2 as a critical mediator by which RTN3-suppressed macrophages promote pNENs metastasis.

## Discussion

TME of most solid tumors is characterized by hypoxia and inflammation, fostering tumor progression and metastasis through complex interactions between TME and cancer cells 17. Notably, the dynamic crosstalk within the TME, particularly between pNEN cells and macrophages, not only induces the reprogramming of macrophages towards an immunosuppressive phenotype conducive to tumor growth but also optimizes the TME to support cancer progression and metastasis 18.

Increasing evidence highlights the role of extracellular vesicles in mediating interactions between cancer cells and macrophages through the transfer of bioactive molecules and genetic material, facilitating communication over distances 19. Our research underscores that exosomes, enriched with miR-4488 and derived from hypoxic cancer cells, can be transferred to macrophages. This transfer leads to enhanced M2 polarization, mediated by the downregulation of RTN3, a direct target of miR-4488, thereby promoting the metastasis of pNEN cells both in vitro and in vivo. This body of work elucidates the pivotal role of exosomes in shaping a hypoxia-driven TME, underlining the significance of extracellular vesicle-mediated communication in tumor biology.

Alterations in key metabolic regulatory processes are critical for the complete activation of M2 macrophages 20. Our comprehensive analysis using RNA sequencing and mass spectrometry revealed that both mRNA levels affected by RTN3 suppression and proteins interacting with RTN3 are predominantly involved in metabolic pathways. FAO, a crucial catabolic mechanism, is instrumental in M2 macrophage activation by supplying ATP for cellular energy demands 21. We have further demonstrated that RTN3 directly interacts with and suppresses the expression of FABP5. This interaction promotes the breakdown of lipid droplets and enhances the oxidation of fatty acids in macrophages through the upregulation of genes associated with FAO, notably CPT1α, CPT2, and ACADL. Intriguingly, our prior research has illustrated that elevated levels of FABP5 in pNEN cells enhance tumor proliferation and metastasis 22. Conversely, FABP5 overexpression within the TME can negate the pro-metastatic effects of RTN3-induced M2 macrophage polarization. This observation suggests that FABP5 exerts diverse roles across different cell types and TME components, meriting further exploration to fully understand its multifaceted impact on tumor biology.

Recent research has implicated the PI3K/AKT/mTOR pathway in the activation of M2 macrophages, both directly and indirectly 23. It has been documented that PI3K activates mTOR complex 2 (mTORC2), which in turn phosphorylates AKT, a critical step in M2 polarization. Certain factors that promote M2 polarization, such as macrophage colony-stimulating factor (M-CSF) and interleukin-4 (IL-4), depend on the activation of the PI3K/AKT/mTOR pathway alongside metabolic reprogramming 24. Our RNA sequencing analysis reveals that the loss of RTN3/FABP5 is inversely associated with the constitutive activation of the PI3K/AKT/mTOR signaling pathway. This suggests that the presence of RTN3/FABP5 may inhibit the activation of this pathway, thereby inducing M2 polarization. This finding underscores the complex regulatory mechanisms involving RTN3/FABP5 in modulating macrophage polarization via the PI3K/AKT/mTOR signaling pathway.

M2-polarized macrophages are pivotal in the regulation of tumor proliferation and metastasis through the secretion of a significant quantity of cytokines into TME 25. These cytokines are crucial in cancer cell biology, facilitating the development and progression of various human malignancies. Our research supports this concept, demonstrating that macrophages induced to adopt an M2 phenotype by exosomal miR-4488 from hypoxic pNEN cells can enhance pNEN metastasis by notably increasing the secretion of MMP2. This increase fosters ongoing communication between hypoxic pNEN cells and TAMs, thus cultivating a malignant microenvironment that promotes tumor metastasis. Interestingly, while the loss of FABP5 does not directly augment MMP2 secretion, it can negate the enhanced MMP2 secretion caused by RTN3 knockdown, thereby inhibiting the pro-metastatic influence of RTN3-suppressed macrophages on pNEN cells. From a clinical perspective, our findings reveal that MMP2 inhibitors can significantly curb the metastasis-promoting effects of RTN3 knockdown-induced M2 macrophages both in vitro and in vivo, presenting viable therapeutic avenues.

Patients with pNENs that have metastasized to the liver are typically faced with a poor prognosis and limited therapeutic options, underscoring the urgency for improved treatment strategies 26. Our study sheds light on the mechanisms by which tumor cells induce metabolic and functional changes in macrophages to facilitate their own proliferation and metastasis, a process particularly relevant in the context of liver metastasis and potentially applicable to other tumor types. We emphasize the critical role of exosomes enriched with miR-4488, released by hypoxic tumor cells, in this tumor-macrophage interaction. The downregulation of RTN3/FABP5 in macrophages, as mediated by these exosomes, promotes fatty acid oxidation and activates the PI3K/AKT/mTOR signaling pathway, leading to M2 polarization. This polarization, in turn, results in the increased secretion of MMP2, further facilitating pNENs metastasis.

Our findings suggest that targeting the pathways influenced by miR-4488 or MMP2 in tumor-associated macrophages could disrupt the metabolic and immune suppression interplay between tumors and macrophages, presenting a promising therapeutic approach to combat liver metastasis. This strategy highlights the potential for targeting specific molecular interactions within the tumor microenvironment to alleviate the progression of metastatic disease.

## Supplementary Material

Supplementary figures and tables.

## Figures and Tables

**Figure 8:**
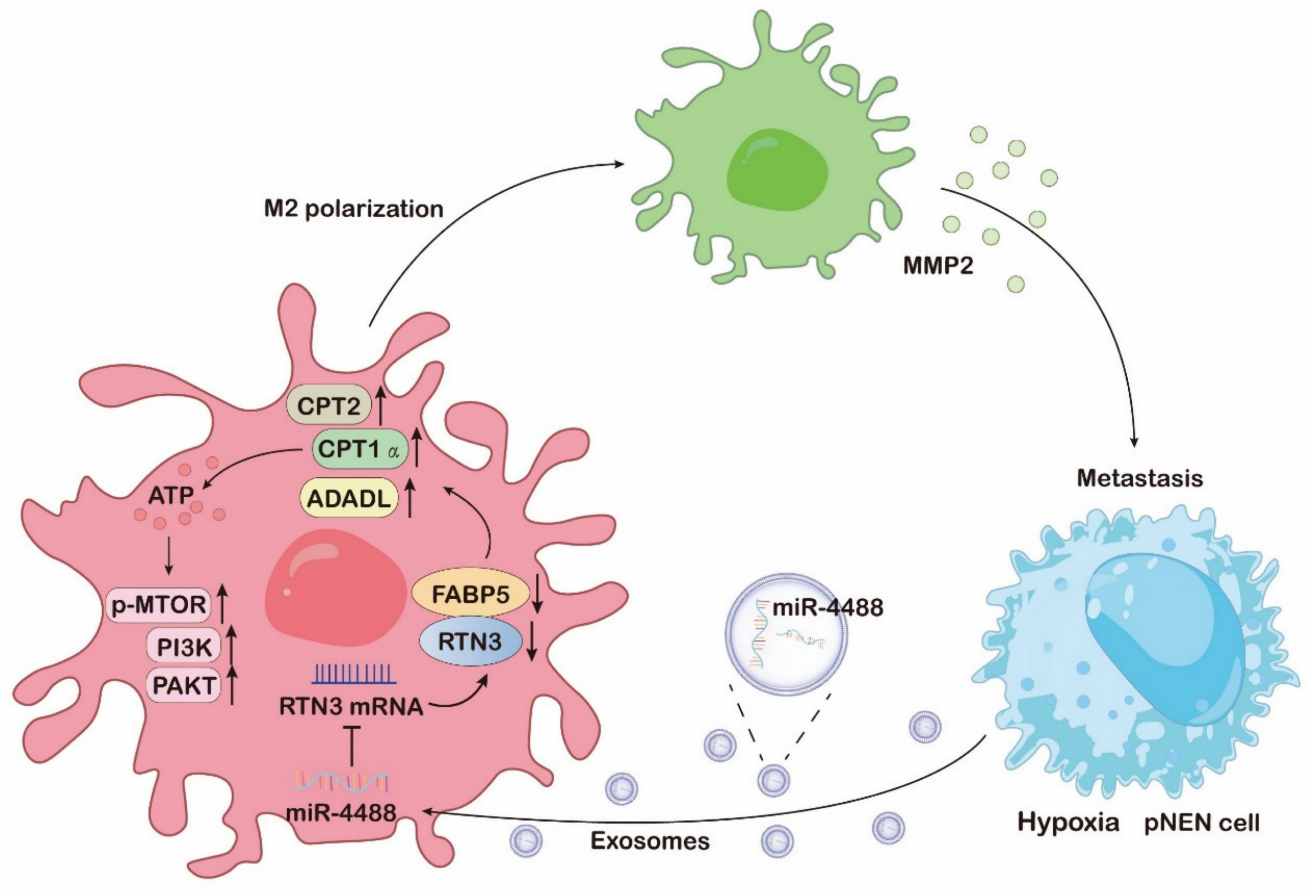
The mechanism diagram of hypoxic pNENs tumor-derived exosomal miR-4488 on inducing macrophage M2 polarization to promote liver metastasis of pNENs through RTN3/FABP5 mediated fatty acid oxidation.

## Abbreviations

TAMs: Tumor-associated macrophages
TME: Tumor microenvironment
pNEN: Pancreatic neuroendocrine neoplasm
EMT: Epithelial-to-mesenchymal transition
H&E: Haematoxylin and eosin
IHC: Immunohistochemical
3'UTR: 3' untranslated region
KEGG: Kyoto Encyclopedia of Genes and Genomes
FAO: Fatty acid oxidation
MMP2: Matrix metalloproteinase-2
