# Supplementary material for: Hypoxic tumor-derived exosomal miR-4488 induces macrophage M2 polarization to promote liver metastasis of pancreatic neuroendocrine neoplasm through RTN3/FABP5 mediated fatty acid oxidation
Source: Int J Biol Sci. 2024 Jun 3;20(8):3201–18. doi: 10.7150/ijbs.96831 (PMC11186367; doi:10.7150/ijbs.96831)
Supplement: Supplementary file 1 — Supplementary figures and tables. [file ijbsv20p3201s1.pdf]

Supplementary Figure 1

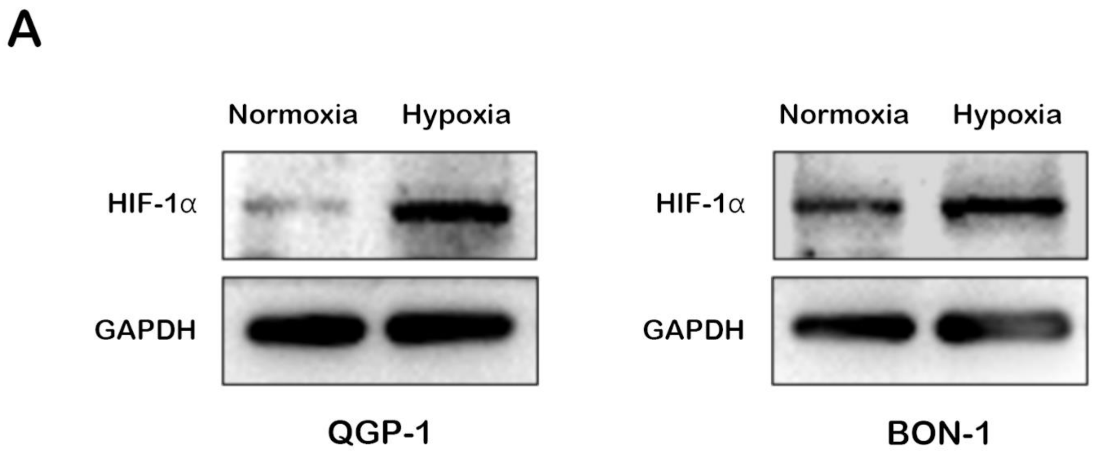

(A) Western blot showed expression of HIF-1α after pNEN cells being cultured under nomoxia or hypoxia.

Supplementary Figure 2

A

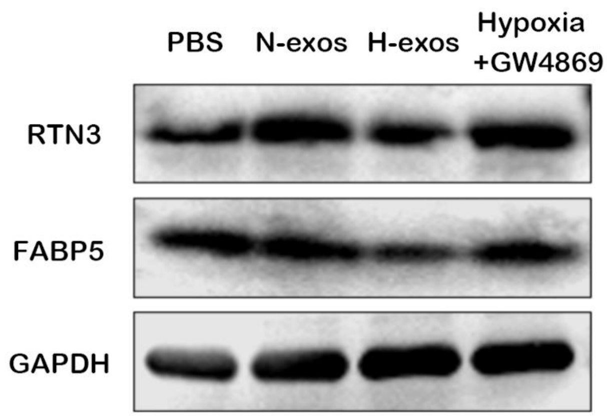

(A) Western blot showed expression of RTN3 and FABP5 after PMA-treated THP-1 cells being incubated with PBS, QGP-1-N-exos, QGP-1-H-exos or exosomes from QGP-1 cells treated with GW4869 under hypoxia.

Supplementary Figure 3

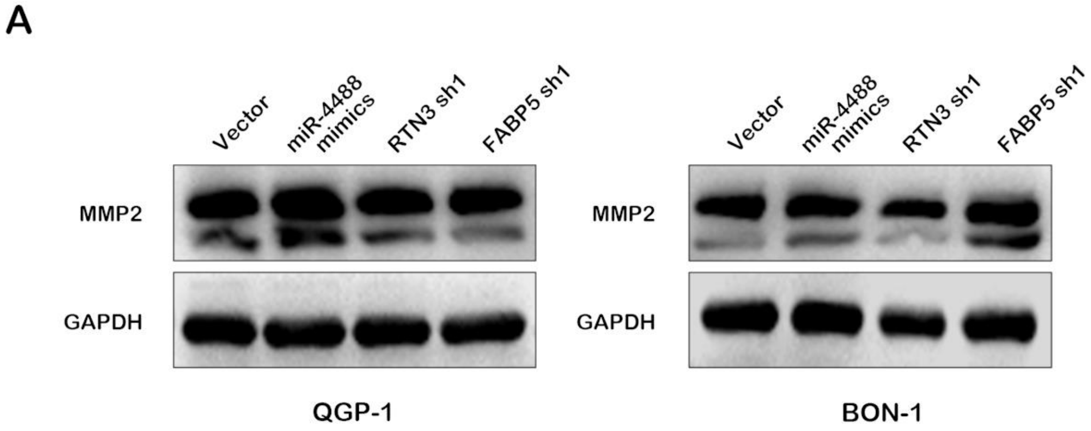

(A) Western blot results for the expression of MMP2 in macrophages transfected with miR-4488 mimics, RTN3 sh1 or FABP5 sh1 plasmids.

Supplementary Table1 Primers of genes

Gene names	Sequence (5'-3')
CD163 forward	TTTGTCAACTTGAGTCCCTTCAC
CD163 reverse	TCCCGCTACACTTGTTTTCAC
IL-10 forward	GACTTTAAGGGTTACCTGGGTTG
IL-10 reverse	TCACATGCGCCTTGATGTCTG
VEGFA forward	AGGGCAGAATCATCACGAAGT
VEGFA forward	AGGGTCTCGATTGGATGGCA
TGF-β forward	GGCCAGATCCTGTCCAAGC
TGF-β reverse	GTGGGTTTCCACCATTAGCAC
TNF-α forward	CTGAACTTCGGGGTGATCGG
TNF-α reverse	GGCTTGTCACTCGAATTTTGAGA
iNOS forward	TTCAGTATCACAACCTCAGCAAG
iNOS reverse	TGGACCTGCAAGTTAAAATCCC
CPT1α forward	CGTTCACGTTTGTTGTCTTC
CPT1α reverse	GACATGACGTACTCCCAAAG
CPT2 forward	CAGCTACCACTGACTCTACT
CPT2 reverse	TTCATGGTGGCATCAAACT
PPARα forward	ATCATGGAACCCAAGTTTGA
PPARα reverse	CGATCTCCACAGCAAATGA
ACAA2 forward	GCTAATGATGCTGGCTACTT
ACAA2 reverse	CTGTTCCAGGGTGGTTTG
ACAD9 forward	CAAGAAGCACTACATCCTCAA
ACAD9 reverse	CAGAATCAACGACCTCAGTC
ACADL forward	CCACAGGAAAGGCTGTTAAT
ACADL reverse	CTGTAGGTGAGCAACTGTTT
MMP2 forward	TACAGGATCATTGGCTACACACC
MMP2 reverse	GGTCACATCGCTCCAGACT
MMP7 forward	GAGTGAGCTACAGTGGGAACA
MMP7 reverse	CTATGACGCGGGAGTTTAACAT
MMP9 forward	TGTACCGCTATGGTTACACTCG
MMP9 reverse	GGCAGGGACAGTTGCTTCT
CSF-1 forward	AGACCTCGTGCCAAATTACATT
CSF-1 reverse	AGGTGTCTCATAGAAAGTTCGGA
EGF forward	TGGATGTGCTTGATAAGCGG
EGF reverse	ACCATGTCCTTTCCAGTGTGT
GAPDH forward	GGAGCGAGATCCCTCCAAAAT

Supplementary Table2 Antibody information

Antibody	Company	Catalogue	Dilution ratio
GAPDH	Proteintech	60004-1-Ig	1：5000
TSG101	Proteintech	28283-1-AP	1：2000
CD63	Proteintech	25682-1-AP	1：500
Calnexin	Proteintech	10427-2-AP	1：1000
RTN3	Proteintech	12055-2-AP	1：2000
FABP5	Proteintech	12348-1-AP	1：3000
CPT1α	Proteintech	15184-1-AP	1：1000
CPT2	Proteintech	26555-1-AP	1：1000
ACADL	Proteintech	17442-1-AP	1：1000
Goat Anti-Mouse IgG	Proteintech	CW0102S	1：5000
Goat Anti-Rabbit IgG	Proteintech	CW0103S	1：2000
P-mTOR	Proteintech	67778-1-Ig	1：5000
mTOR	Proteintech	66888-1-Ig	1：5000
PI3K	Proteintech	67121-1-Ig	1：5000
P-AKT	Proteintech	66444-1-Ig	1：5000
AKT	Proteintech	60203-2-Ig	1：5000
MMP2	Proteintech	10373-2-AP	1：2000

Supplementary Table3 Sequences for short hairpin targets, siRNAs, miRNA mimics and inhibitor

Gene names	Target sequence (5’-3’)
RTN3 sh1	GCTGATGACAGATTCACTTTG
RTN3 sh2	GCAAGGCAATATGCAGAAACA
FABP5 sh1	GGCGCCTGGTGGACAGCAAAG
FABP5 sh2	GCAATGGCCAAGCCAGATTGT
siMMP2-1 (sense)	GUGAGAUCUUCUUCUUCAA
siMMP2-1 (antisense)	UUGAAGAAGAAGAUCUCAC
siMMP2-2 (sense)	GCCUUCAGCUCUACAGCUA
siMMP2-2 (antisense)	UAGCUGUAGAGCUGAAGGC
mimics NC (sense)	UUCUCCGAACGUGUCACGU
mimics NC (antisense)	GUGACACGUUCGGAGAAUU
miR-4488 mimics (sense)	AGGGGGCGGGCUCCGGCG
miR-4488 mimics (antisense)	CCGGAGCCCGCCCCCUUU
inhibitor NC (with full chain methoxyl modification)	(mA)(mC)(mG)(mU)(mG)(mA)(mC)(mA)(mC) (mG)(mU)(mU)(mC)(mG)(mA)(mG)(mA)(mA)
miR-4488 inhibitor (with full chain methoxyl modification)	(mC)(mG)(mC)(mC)(mG)(mG)(mA)(mG)(mC) (mC)(mC)(mG)(mC)(mC)(mC)(mC)(mC)(mU)
